# Does Medical School Prestige Impact Match Rates in Competitive Specialties? A Retrospective Analysis

**DOI:** 10.7759/cureus.85524

**Published:** 2025-06-07

**Authors:** Nathan Cuttica, Alexander Keith, Stephen E Hedberg, Nicholas Sciarretti, Jessica Ralph, Peter S Hedberg

**Affiliations:** 1 College of Medicine, Northeast Ohio Medical University, Rootstown, USA; 2 Department of Clinical and Health Psychology, University of Florida, Gainesville, USA; 3 Department of Surgery, Harvard Medical School, Boston, USA

**Keywords:** competitive specialties, medical school, national residency match program (nrmp) match, residency match, specialty match rates

## Abstract

Matching into a competitive specialty has historically required extensive research, leadership, and extracurricular activities. Since the United States Medical Licensing Examination (USMLE) Step 1 exam has changed to a pass-fail grading system, it has been questioned whether medical school ranking will become a significant factor in matching into competitive medical specialties. To our knowledge, this is among the first studies to examine competitive specialty match trends following the transition to pass/fail Step 1 scoring.

High-tier and low-tier medical schools were determined using the U.S. News and World Report 2024 Medical School Rankings list, and the 20 highest- and 20 lowest-ranked US-based allopathic medical schools were selected. Medical school competitiveness was determined using the mean USMLE Step 2 clinical knowledge (CK) score, mean number of research abstracts, publications, and presentations, percent of applicants that matched into said specialty, and percent of matched students that were Alpha Omega Alpha (AOA) honors society members. The most competitive specialties were determined to be dermatology, neurological surgery, plastic surgery, orthopedic surgery, and otolaryngology. Match rates into competitive specialties were compared at high- and low-tier medical schools. Secondary analysis eliminated schools that did not have an affiliated residency program, termed a “home program”, to examine this as a potential confounder.

High-tier medical schools demonstrated statistically greater match rates into dermatology (p=0.046), neurological surgery (p=0.008), otolaryngology (p=0.008), and competitive specialties overall (p=0.006) when compared to low-tier medical schools. A difference was not noted for plastic surgery (p=0.308) and orthopedic surgery (p=0.809) match rates. However, when accounting for whether schools had affiliated residency programs, these differences were no longer statistically significant, highlighting the potential importance of home programs over school prestige alone.

Higher-tier medical schools placed more students into competitive specialties, specifically dermatology, neurosurgery, and otolaryngology, when compared to lower-tier medical schools. Thus, attending a highly ranked allopathic medical school may serve as a significant benefit when applying to some competitive specialties. However, after eliminating high- and low-tier medical schools without an affiliated residency program, no statistical significance was observed for dermatology, neurosurgery, plastic surgery, orthopedic surgery, otolaryngology, or overall. Thus, medical school ranking may be less important than attending a school with a home program when applying for one of the five competitive specialties examined.

## Introduction and background

Introduction

Matching into a competitive medical specialty has always required extensive research, leadership, extracurricular activities, and an excellent score on standardized exams [1]. Historically, the United States Medical Licensing Examination (USMLE) Step 1, a standardized exam taken in the second year of medical school, was a major predictor of medical student competitiveness in the residency match [2]. In January 2022, the USMLE transitioned the Step 1 examination from a numeric score to a pass/fail outcome only [3], making the class of 2024 the first class to match into residency without a numerical Step 1 score.

While adapting Step 1 from numeric scores to being pass/fail has its merit, it has sparked concern across medical student populations. Despite being familiar with the need to be involved in extracurricular activities, this change has prompted an increase in students’ research and overall extracurricular engagement that has not been seen before [4,5]. Moreover, speculation has arisen regarding whether medical school prestige will become a significant predictive factor in determining residency competitiveness [6].

Previous literature has identified the importance of research experiences, extracurricular activities, and an above-average USMLE Step 1 score for a competitive residency application [1,7,8]. In the wake of the Step 1 scoring transition, students’ commitment to extracurricular endeavors has increased, and questions regarding extraneous factors that might impact residency match have evolved, including medical school prestige. Thus, this study aims to examine the relationship between match rates into competitive specialties at high-tier versus low-tier medical schools for class of 2024 graduates.

Materials & methods

Study Design

The medical school tier was adapted using the public U.S. News and World Report 2024 Medical School Rankings list [9]. The U.S. News and World Report ranks medical colleges by surveying all participating Liaison Committee on Medical Education (LCME) accredited medical schools. Out of 154 participating accredited medical schools, high-tier medical schools were defined as the top 20 US-based allopathic schools. Low-tier medical schools were defined as the 20 lowest-ranked US-based allopathic schools. While it affects generalizability, the 20 highest- and lowest-ranked medical schools were purposely selected to best examine the dichotomy of medical school rankings. Competitiveness of medical specialties was determined using The National Resident Matching Program (NMRP) 2024 Charting Outcomes [10], a publicly available report that evaluated match statistics for all 22 specialties.

Data Collection

While no standard exists for defining specialty competitiveness, we designed a scale that draws on variables widely reported as key match factors, including USMLE Step 2 CK mean score [4], mean number of research abstracts, publications, and presentations [4], percentage of students that were AOA honors society members [11], as well as percentage of applicants that matched. In this scoring system, one point was attributed to the medical specialty with the greatest mean USMLE Step 2 CK score, two points to the medical specialty with the second greatest mean USMLE Step 2 CK score, etc. Given the novelty of this study, the analytic approach was adapted to include multiple factors that determined residency competitiveness without a numerical Step 1 score. This scoring scale was completed for all factors listed above and for all 22 medical specialties listed in the NMRP’s report (Table 1).

**Table 1:** Medical specialties ranked based on mean USMLE Step 2 CK score, mean number of research abstracts, publications, and presentations, percentage of applicants that matched, and percentage of students that were AOA honors society members. USMLE: United States Medical Licensing Examination; CK: clinical knowledge; AOA: Alpha Omega Alpha

Rank	Specialty	Points
1	Dermatology	7
2	Plastic Surgery	10
2	Neurological Surgery	10
4	Orthopedic Surgery	11
5	Otolaryngology	17
6	General Surgery	27
7	Interventional Radiology	28
8	Diagnostic Radiology	32
9	Obstetrics and Gynecology	34
10	Anesthesiology	39
10	Internal Medicine/Pediatrics	39
10	Vascular Surgery	39
13	Radiation Oncology	41
14	Physical Medicine and Rehabilitation	46
15	Internal Medicine	50
16	Child Neurology	51
17	Neurology	53
18	Pathology	61
18	Psychiatry	61
20	Pediatrics	66
20	Emergency Medicine	66
22	Family Medicine	72

The five most competitive specialties, dermatology, plastic surgery, neurological surgery, orthopedic surgery, and otolaryngology, were further evaluated, as it is hypothesized that competitive specialties will most likely be impacted by the Step 1 scoring change; thus, they were included for final analyses.

Statistical Analysis

Using each institution’s public 2024 match records, the percentage of students who matched into dermatology, plastic surgery, neurological surgery, orthopedic surgery, and otolaryngology at their respective medical schools were summated and compared between high-tier and low-tier allopathic medical schools. Data were analyzed using two-sample t-tests with unequal variances. All statistical analyses were performed using Microsoft Excel.

Using the same sample of medical schools identified as high-tier and low-tier, those without a home residency program were excluded from further analysis. This was performed to evaluate a potentially confounding relationship between medical schools being affiliated with a residency program and the match rates into that respective specialty, as it has been demonstrated that medical schools with an affiliated “home program” may produce greater match rates due to more opportunities for early exposure and mentorship [12]. Following the implementation of exclusion criteria, the sample size of participating schools for each specialty varied. Numerous high-tier medical schools were identified as having a dermatology (n=19), plastic surgery (n=17), neurological surgery (n=18), orthopedic surgery (n=19), and otolaryngology (n=18) residency. Low-tier medical schools also had affiliated dermatology (n=11), neurological surgery (n=9), plastic surgery (n=7), orthopedic surgery (n=15), and otolaryngology (n=10) residency programs. The data collection process and statistical analysis were performed following the same procedure.

## Review

Results

At high-tier allopathic medical schools, 2.93% (SD=1.9) of students matched into dermatology, compared to 1.69% (SD=1.6) at low-tier medical schools (p=.046) (Table 2).

**Table 2:** Percent of matched students at high-tier and low-tier medical schools in each competitive specialty and total percent matched into competitive specialties. ± represents 95% confidence intervals (CI). Statistical significance is represented by p<0.05>.

Specialty	High Tier (% of class)	Low Tier (% of class)	p-value
Dermatology	2.93 ± 0.8	1.69 ± 0.7	0.046
Neurosurgery	1.75 ± 0.6	0.68 ± 0.4	0.008
Plastic Surgery	1.22 ± 0.4	0.95 ± 0.3	0.308
Orthopedic Surgery	3.81 ± 1.0	3.65 ± 0.8	0.809
Otolaryngology	2.53 ± 0.5	1.39 ± 0.5	0.008
Total	12.15 ± 1.9	8.37 ± 1.4	0.006

At high-tier medical schools, 1.7% (SD=1.3) of students matched into neurological surgery, compared to 0.68% (SD=0.9) at low-tier medical schools (p=.008). For integrated plastic surgery residency programs, 1.22% (SD=0.9) of students at high-tier schools matched, compared to 0.95% (SD=0.7) at lower-ranked medical schools (p=.308). For orthopedic surgery, high-tier medical schools averaged 3.81% (SD=2.2) of their class matched and low-tier schools averaged 3.65% (SD=1.7) (p=0.809). Match rates into otolaryngology at high-tier schools were 2.53% (SD=1.2) and 1.39% (SD=1.1) at low-tier medical schools (p=0.008). Overall, top-ranked medical schools placed 12.5% (SD=4.4) of their class into one of these five specialties, compared to 8.37% (SD=3.2) for lower-ranked medical schools (p=0.006).

High- and low-tier medical schools were excluded if they did not have an affiliated residency program to examine the role of “home programs” in match rates. Following implementation of exclusion criteria, 2.93% (SD=1.9) of students at high-tier allopathic medical schools matched into dermatology, compared to 1.81% (SD=1.4) at low-tier medical schools (p=0.328) (Table 3).

**Table 3:** Percentage of matched students at high-tier and low-tier medical schools with home residency programs. ± represents 95% confidence intervals (CI). Statistical significance is represented by p<0.05>

Specialty	High Tier (% of class)	Low Tier (% of class)	p-value
Dermatology	2.93 ± 0.9	1.81 ± 1.0	0.328
Neurosurgery	1.81 ±.07	0.97 ± 0.7	0.132
Plastic Surgery	1.17 ± 0.4	1.06 ± 0.5	0.764
Orthopedic Surgery	3.65 ± 1.0	3.58 ± 0.9	0.928
Otolaryngology	2.53 ± 0.6	1.72 ± 0.7	0.104
Total	12.09 ± 0.8	9.14 ± 0.9	0.313

High-tier medical schools with an affiliated neurosurgery residency placed 1.81% (SD=1.4) into neurosurgery, whereas low-tier medical schools placed 0.97% (SD=1.0) into neurosurgery residency (p=0.132). At higher-ranked medical schools, 1.17% (SD=0.9) of students matched into an integrated plastic surgery residency program, compared to 1.06% (SD=0.7) at lower-ranked medical schools (p=0.764). For orthopedic surgery, high-tier medical schools averaged 3.65% (SD=2.1) of their class matched and low-tier schools averaged 3.58% (SD=1.8) (p=0.928). For otolaryngology, 2.53% (SD=1.2) of students at high-tier allopathic medical schools matched compared to 1.72% (SD=1.2) at low-tier medical schools (p=0.104). Overall, top-ranked medical schools with an affiliated home residency program placed 12.09% (SD=1.77) of their class into one of these five specialties, compared to 9.14% (SD=1.74) for lower-ranked medical schools (p=0.313).

Discussion

Statistical analyses revealed that a greater percentage of students at higher-tier medical schools matched into competitive specialties when compared to low-tier medical schools (p=0.006), specifically in dermatology (p=0.046), neurological surgery (p=0.008), and otolaryngology (p=0.008). Findings for dermatology [13], neurological surgery [14], and otolaryngology [15] are comparable to data collected prior to the elimination of a USMLE Step 1 numerical score. Comparing the groups yielded no difference in percentage of students placed into plastic surgery (p=0.308) and orthopedic surgery (p=0.809). These findings are contradictory to past evaluations of match statistics for plastic surgery [16] and orthopedic surgery [17]. Thus, with the recent elimination of a numerically scored USMLE Step 1 examination, match rates into competitive specialties may affect both high- and low-tier medical schools.

However, after excluding medical schools without an affiliated residency program, competitive specialty match rates were not statistically significant between high- and low-tier medical schools (p=0.313). Additionally, match rates for high- and low-tier medical schools in dermatology (p=0.328), neurological surgery (p=0.132), plastic surgery (p=0.764), orthopedic surgery (p=0.928), and otolaryngology (p=0.104) were similar. This is comparable to previous literature indicating the importance of home programs on match rates, due to potential opportunities including more exposure to certain fields and early mentorship [12,18].

Limitations of this study included a small sample size (n=20). While small sample size does limit the power of this study, the 20 highest- and lowest-ranked medical schools were purposely selected to best examine the dichotomy of medical school rankings; however, this also limited broad generalizability. Another limitation was that several prestigious medical schools recently opted not to be included in the U.S. News and World Report Medical School Rankings list. Therefore, multiple highly ranked medical schools were excluded from the study. Inclusion of these universities could potentially have strengthened findings of match rates at high- and low-tier medical schools.

To our knowledge, this is the first study examining match rates into competitive specialties dependent on medical school prestige following the elimination of a numerical USMLE Step 1 score. Future research would benefit from further exploration of a broader sample that includes mid-tier medical schools to enhance the robustness and generalizability of the study, as well as examining osteopathic medical school match rates. Also, examining factors that lead to greater match rates at schools with affiliated residency programs, such as research opportunities, mentorship, and networking opportunities.

## Conclusions

To our knowledge, this is among the first studies to examine competitive specialty match trends following the transition to pass/fail Step 1 scoring. Higher-tier medical schools placed a higher percentage of students into competitive specialties, specifically dermatology, neurosurgery, and otolaryngology, when compared to lower-tier medical schools. Thus, attending a highly ranked allopathic medical school may serve as a significant benefit when applying to some competitive specialties.

However, when comparing high- and low-tier medical schools with an affiliated residency program, no statistical significance was observed for dermatology, neurosurgery, plastic surgery, orthopedic surgery, or otolaryngology. Thus, medical school ranking may be less important than attending a school with a home program when applying for one of the five competitive specialties examined.
